# Association between albuminuria and arterial stiffness measured by brachial-ankle pulse wave velocity among Chinese population: A retrospective study

**DOI:** 10.1097/MD.0000000000047855

**Published:** 2026-02-28

**Authors:** Wei-Chung Yeh, Wen-Cheng Li, Mei-Wen Wang, Chen-Wei Hsu, Yu-Chung Tsao, Yu-Hsiang Lin, Jau-Yuan Chen

**Affiliations:** a Department of Family Medicine, Chang Gung Memorial Hospital, Keelung Branch, Keelung, Taiwan; b Department of Family Medicine, Chang-Gung Memorial Hospital, Linkou Branch, Taoyuan, Taiwan; c College of Medicine, Chang-Gung University, Taoyuan, Taiwan; d Department of Health Management, Xiamen Chang-Gung Hospital, Xiamen, China; e Department of Occupational Medicine, Chang Gung Memorial Hospital Linkou, Taoyuan, Taiwan; f Master of Science Degree Program in Innovation for Smart Medicine, Chang Gung University, Taoyuan, Taiwan; g College of Life Sciences and Medicine, National Tsing Hua University, Hsinchu, Taiwan; h Department of Urology, Chang-Gung Memorial Hospital, Linkou Branch, Taoyuan, Taiwan.

**Keywords:** albuminuria, arterial stiffness, brachial-ankle pulse wave velocity, cardiovascular risk, chronic kidney disease, endothelial dysfunction

## Abstract

Albuminuria, a hallmark of early kidney damage, has been increasingly recognized for its association with cardiovascular morbidity. Similarly, arterial stiffness (quantified by brachial-ankle pulse wave velocity [baPWV]) serves as an indicator of vascular health. The 2 conditions may be interconnected through shared pathophysiological mechanisms, with endothelial dysfunction playing a central role. This retrospective study aimed to investigate the association between albuminuria and arterial stiffness measured by baPWV in a Chinese health checkup population. We retrospectively analyzed data from 5025 adults who underwent health examinations between 2013 and 2014. Albuminuria was defined as an albumin-to-creatinine ratio ≥30 mg/g, and arterial stiffness as baPWV ≥1400 cm/s. Logistic regression models were used to evaluate the association between baPWV and albuminuria, adjusting for confounders including age, sex, and metabolic indicators. Participants with albuminuria exhibited significantly higher body mass index, waist circumference, blood pressure, triglycerides, fasting plasma glucose, and baPWV values compared to those without albuminuria (*P* < .001). Elevated baPWV was independently associated with increased odds of albuminuria (adjusted odds ratio 1.66, 95% confidence interval: 1.18–2.33, *P* = .004). Waist-to-height ratio, mean arterial pressure, and fasting glucose were also significant predictors of albuminuria. Albuminuria is independently associated with increased arterial stiffness in a Chinese health checkup population. Prospective studies are warranted to explore causality and therapeutic implications.

## 1. Introduction

Chronic kidney disease (CKD) is a global health concern, with albuminuria and glomerular filtration rate (GFR) serving as its principal markers. Albuminuria, the abnormal excretion of albumin in urine, is quantitatively defined by an albumin-to-creatinine ratio (ACR) exceeding 30 mg/g in spot urine samples. Its clinical significance extends beyond nephrology, as recent meta-analyses have established its association with an increased risk of cardiovascular morbidity.^[1,2]^ The pathogenesis of albuminuria is attributed to elevated glomerular pressure and the compromised integrity of the glomerular capillary barrier, with endothelial dysfunction identified as a central etiological factor.^[3,4]^

Arterial stiffness, characterized by a decrease in arterial elasticity, is widely recognized as a precursor to various cardiovascular complications, such as coronary heart disease, stroke, and peripheral arterial disease.^[5,6]^ The multifactorial etiology of arterial stiffness includes traditional cardiovascular risk factors such as aging, smoking, obesity, hypertension, and diabetes.^[7,8]^

The carotid-femoral pulse wave velocity (PWV) is a well-established method for evaluating arterial stiffness. However, the brachial-ankle PWV (baPWV) has gained recognition for its practicality and ease of use in clinical settings, providing a reliable surrogate for assessing arterial stiffness.^[9]^ Recent community-based data from Beijing further demonstrated that both carotid-femoral PWV and baPWV were significantly associated with albuminuria, supporting the use of baPWV as a practical marker of vascular damage in population-based studies.^[10]^ Conducted in the context of these findings, this study aimed to explore the link between albuminuria and baPWV within a cohort from China. We investigated the interconnectedness of kidney and vascular diseases, focusing on endothelial dysfunction as a key contributor to both renal impairment and arterial stiffness. Accordingly, the central hypothesis was that higher baPWV, as a marker of arterial stiffness, would be independently associated with the presence of albuminuria, even after adjustment for conventional cardiovascular and renal risk factors.

## 2. Methods

### 2.1. Subjects

This study retrospectively collected the medical examination records of Chinese adults (aged ≥ 18 years) who underwent health checkups at the Health Management Center in China, from 2013 to 2014. Data were collected by trained nurses using a standardized questionnaire that included questions on demographics, disease history, medication use, and physiological conditions (including pregnancy and fasting time) at the beginning of the examination. This standardized questionnaire was developed by our institution, has been routinely used in the health examination program, and its items are derived from standard clinical practice, although the instrument as a whole has not undergone formal validation. This was followed by a detailed physical examination and fasting blood collection for laboratory measures. All information was entered into a centralized electronic database under regular strict quality control monitoring.

We excluded subjects with insufficient information or who met any of the following exclusion criteria: presence of urinary tract infection; failure to fast for at least 12 hours prior to blood sampling; pregnancy; diagnosis of chronic diseases known to significantly affect metabolism, including thyroid disorders, tumors, recent surgery, chronic hepatitis, liver cirrhosis, hypothalamic disorders, and adrenal gland disorders, among others. A total of 5025 individuals (2830 males and 2195 females) were included in the final analysis. The study was approved by the Institutional Review Board and was conducted in accordance with the Helsinki Declaration.

**Figure 1.**
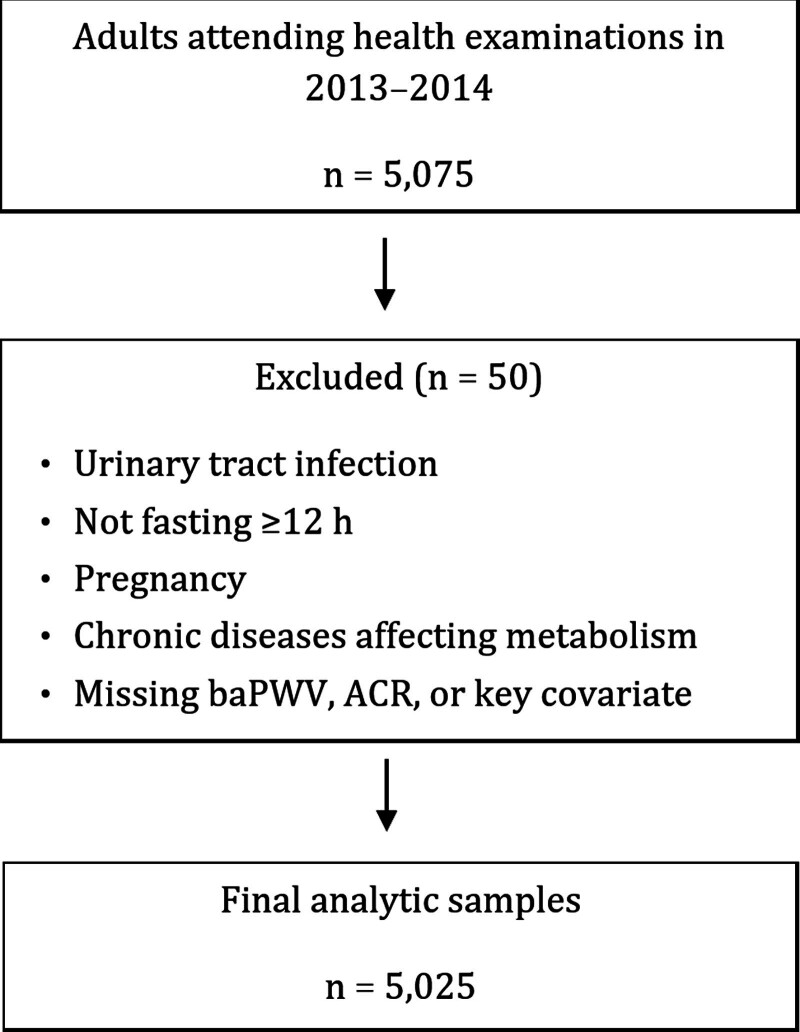
Flowchart of participant selection from the initial health examination cohort to the final analytic sample.

We excluded subjects with insufficient information (including missing data on baPWV, ACR, or key covariates) or who met any of the following exclusion criteria: presence of urinary tract infection; failure to fast for at least 12 hours prior to blood sampling; pregnancy; diagnosis of chronic diseases known to significantly affect metabolism, including thyroid disorders, tumors, recent surgery, chronic hepatitis, liver cirrhosis, hypothalamic disorders, and adrenal gland disorders, among others. Thus, the final sample included 5025 individuals (2830 males and 2195 females) who met the eligibility criteria and had complete data (Fig. 1). The study was approved by the Institutional Review Board and was conducted in accordance with the Helsinki Declaration.

### 2.2. Measurements

Body height and weight were measured using calibrated meters and scales in accordance with a standard protocol. Waist circumference was measured midway between the lowest rib and the iliac crest. Blood pressure was measured 3 times after a 10-minute rest, with the subject seated and using an automated sphygmomanometer placed on the subject’s right arm. Mean arterial pressure (MAP) was estimated using the following equation: 2/3 × diastolic blood pressure (DBP) + 1/3 × systolic blood pressure (SBP). Subjects were instructed to avoid high-fat diets or alcohol consumption for at least 24 hours prior to phlebotomy. Blood samples were collected after a minimum of 12 hours of fasting and were processed according to the hospital’s laboratory standard operating procedures, which are accredited by the College of American Pathologists. Venous blood was drawn between 8:00 am and 11:00 am and stored in a 4 °C refrigerator prior to analysis.

Fasting plasma glucose (FPG), total cholesterol (TC), triglycerides (TG), high-density lipoprotein cholesterol (HDL-C), and serum creatinine were measured using a biochemical auto-analyzer (DxC 800, Beckman Coulter UniCel DxC SYNCHRON, Galway, Ireland). Urinary albumin and creatinine concentrations from spot urine samples were measured using the UniCel DxC 800 MA & CREA Reagent system. ACR were then calculated for all participants.

### 2.3. Assessment of baPWV

BaPWV was measured using an ABI-form device (VP1000, Colin Co. Ltd, Komaki, Japan), which allows simultaneous measurement of SBP and pulse waves at the brachial and posterior tibial arteries on both sides. This method is highly efficient and reproducible, and has been validated as a reliable marker of vascular damage.^[11]^ The average of the left and right baPWV values was used for subsequent analysis.

### 2.4. Definitions

Body mass index (BMI) was calculated as weight in kilograms divided by the square of height in meters. The estimated GFR was calculated based on serum creatinine (1 mg/dL = 88.4 µmol/L) using the Modification of Diet in Renal Disease equation, with adjustments for sex, age, and race.^[12,13]^ The equation was as follows:


$$\begin{array}{l} eGFR\left(mL/min/1.73\;m^{2}\right)=175\;\times SCr^{-1.234}\times Age^{-0.179}\left(\times 0.79\;\;if\;female\right). \end{array}$$

Based on the recommended cutoffs for ACR,^[14,15]^ albuminuria was defined as ACR ≥30 mg/g Cr. Arterial stiffness was defined as baPWV ≥1400 cm/s, in accordance with previous guidelines and large East Asian cohort studies in which this cutoff has been widely used to indicate increased arterial stiffness and higher cardiovascular risk.^[16,17]^

### 2.5. Statistical analyses

Statistical analyses were performed using SPSS version 22.0 (SPSS Inc., Chicago). Continuous variables were reported as means ± standard deviations, and categorical variables as percentages. Participants were divided into 2 groups based on ACR level. Between-group differences were assessed using independent two-sample *t* tests for continuous variables and Chi-square tests for categorical variables. Logistic regression analysis was conducted to control for potential confounding variables and to evaluate the independent association between albuminuria and baPWV. All statistical tests were two-tailed, and a *P*-value of <.05 was considered statistically significant. In addition, a post hoc power analysis using G*Power indicated that the available sample size provided ample statistical power to detect the observed association at a two-sided α of 0.05.

## 3. Results

Our analysis of 5025 subjects highlighted significant sex-specific differences in various clinical and metabolic parameters. Males showed higher BMI, waist circumference, and baPWV, while females had elevated HDL-C levels. The differences in SBP, DBP, MAP, FPG, TC, TG, and the TG/HDL-C ratio were statistically significant between the sexes (all *P* < .001) (Table 1).

**Table 1:** Basic characteristics of the study subjects (n = 5025).

Variables	Total	Male	Female	*P*-value
	(n = 5025)	(n = 2829)	(n = 2196)	
Age (y/o)	46.88 ± 10.34	46.64 ± 10.19	47.19 ± 10.52	.06
BMI	23.79 ± 3.30	24.38 ± 3.20	23.04 ± 3.27	<.001
WC (cm)	83.53 ± 9.82	86.99 ± 8.83	79.08 ± 9.22	<.001
WHtR	0.51 ± 0.06	0.51 ± 0.05	0.50 ± 0.06	<.001
SBP (mm Hg)	117.73 ± 18.31	119.89 ± 16.92	114.95 ± 19.62	<.001
DBP (mm Hg)	72.40 ± 11.80	75.42 ± 11.49	68.50 ± 11.03	<.001
MAP (mm Hg)	87.51 ± 13.27	90.24 ± 12.77	83.99 ± 13.07	<.001
FPG (mmol/L)	5.30 ± 1.29	5.41 ± 1.46	5.17 ± 1.00	<.001
TC (mmol/L)	5.18 ± 0.97	5.24 ± 0.95	5.09 ± 1.00	<.001
TG (mmol/L)	1.56 ± 1.51	1.84 ± 1.59	1.19 ± 1.30	<.001
HDL-C (mmol/L)	1.32 ± 0.31	1.22 ± 0.27	1.44 ± 0.30	<.001
TG/HDL-C	1.37 ± 1.94	1.70 ± 2.04	0.93 ± 1.72	<.001
Serum Cr (µmol/L)	73.26 ± 14.83	82.69 ± 11.43	61.12 ± 8.53	<.001
eGFR (mL/min/1.73 m^2^)	102.54 ± 10.09	101.01 ± 8.99	104.51 ± 11.04	<.001
baPWV (cm/s)	1334.42 ± 237.62	1357.08 ± 216.66	1305.22 ± 259.30	<.001
ACR (mg/g Cr)	10.91 ± 58.36	11.65 ± 70.95	9.96 ± 36.16	.31
ACR ≧ 30 mg/g Cr (%)	249 (4.96%)	148 (5.23%)	101 (4.60%)	.31

*Notes*: Continuous data are shown as mean ± standard deviation and compared using independent 2 samples *t* test. Categorical data are shown as n (%) and compared using the Chi-square test.

ACR = albumin/creatinine ratio, baPWV = brachial-ankle pulse wave velocity, BMI = body mass index, DBP = diastolic blood pressure, eGFR = estimated glomerular filtration rate, FPG = fasting plasma glucose, HDL-C = high-density lipoprotein cholesterol, MAP = mean arterial pressure, SBP = systolic blood pressure, SCr = serum creatinine, TC = total cholesterol, TG = triglycerides, WC = waist circumstance, WHtR = waist-to-height ratio.

**Figure 2.**
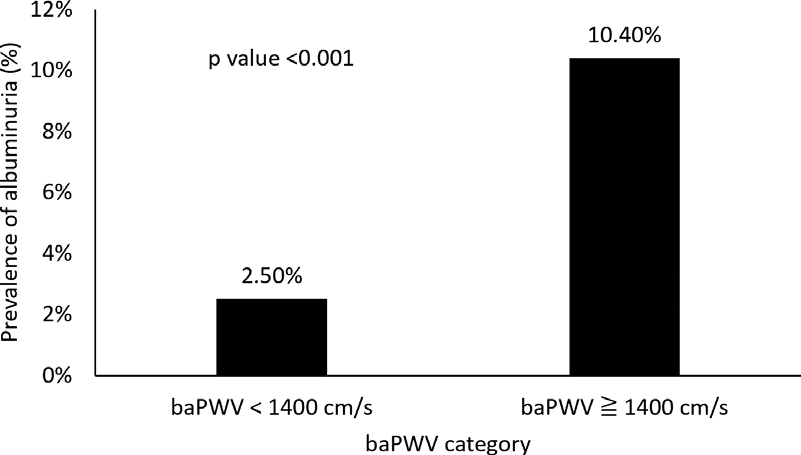
Prevalence of albuminuria according to baPWV category. baPWV = brachial-ankle pulse wave velocity.

When categorized based on albuminuria, participants with an ACR of 30 mg/g or higher showed significantly higher average values for BMI, waist circumference, waist-to-height ratio (WHtR), SBP, DBP, MAP, FPG, TC, and TG, and lower HDL-C levels compared to those with an ACR below 30 mg/g. A significant disparity was also observed in baPWV, with a notably higher prevalence in the albuminuria group (65.46% had baPWV ≥1400 cm/s) compared to those without albuminuria (29.27%, *P* < .001) (Table 2). Consistently, as illustrated in Figure 2, the prevalence of albuminuria was 2.5% among participants with baPWV <1400 cm/s and 10.4% among those with baPWV ≥1400 cm/s (*P* < .001).

**Table 2:** Characteristics of subjects after cross-classification by albuminuria (AU).

Variables	No AU (ACR < 30)	AU (ACR ≧ 30)	*P* value
	(n = 4776)	(n = 249)	
Age (y/o)	46.67 ± 10.25	50.81 ± 11.24	<.001
Men, n (%)	2095 (43.87%)	101 (40.56%)	.31
BMI	23.70 ± 3.22	25.61 ± 4.12	<.001
WC (cm)	83.24 ± 9.64	89.13 ± 11.43	<.001
WHtR	0.51 ± 0.05	0.55 ± 0.07	<.001
SBP (mm Hg)	116.77 ± 17.32	136.13 ± 25.64	<.001
DBP (mm Hg)	71.87 ± 11.30	82.45 ± 16.07	<.001
MAP (mm Hg)	86.84 ± 12.61	100.35 ± 18.15	<.001
FPG (mmol/L)	5.24 ± 1.10	6.52 ± 2.92	<.001
TC (mmol/L)	5.17 ± 0.96	5.37 ± 1.09	.001
TG (mmol/L)	1.52 ± 1.44	2.27 ± 2.34	<.001
HDL-C (mmol/L)	1.32 ± 0.31	1.26 ± 0.31	.002
TG/HDL-C	1.33 ± 1.81	2.18 ± 3.61	<.001
eGFR (mL/min/1.73 m^2^)	102.73 ± 9.97	98.86 ± 11.50	<.001
baPWV (cm/s)	1323.01 ± 227.63	1553.26 ± 309.11	<.001
baPWV ≧ 1400 (cm/s), n (%)	1398 (29.27%)	163 (65.46%)	<.001

*Notes*: Continuous data are shown as mean ± standard deviation and compared using independent 2 samples *t* test. Categorical data are shown as n (%) and compared using the Chi-square test.

ACR = albumin/creatinine ratio, baPWV = brachial-ankle pulse wave velocity, BMI = body mass index, DBP = diastolic blood pressure, eGFR = estimated glomerular filtration rate, FPG = fasting plasma glucose, HDL-C = high-density lipoprotein cholesterol, MAP = mean arterial pressure, SBP = systolic blood pressure, SCr = serum creatinine, TC = total cholesterol, TG = triglycerides, WC = waist circumstance, WHtR = waist-to-height ratio.

**Figure 3.**
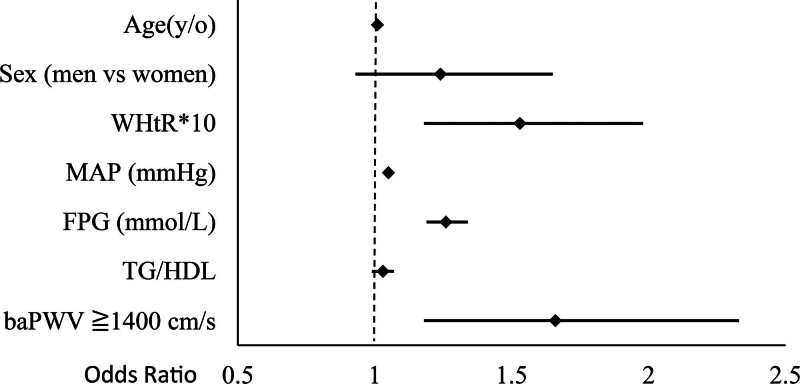
Multivariable-adjusted odds ratio for albuminuria.

Logistic regression analysis identified WHtR, MAP, and FPG as significant independent indicators of albuminuria. For every 0.1 increase in WHtR, the likelihood of albuminuria increased by 53% (adjusted odds ratio [OR] 1.53, 95% confidence interval: 1.18–1.98, *P* = .001), indicating a strong correlation between central obesity and renal risk. Increases in MAP and FPG were associated with 5% (adjusted OR 1.05, *P* < .001) and 26% (adjusted OR 1.26, *P* < .001) higher odds of albuminuria, respectively. Moreover, baPWV values of 1400 cm/s or more were significantly associated with albuminuria, with a 66% increase in the odds (adjusted OR 1.66, 95% confidence interval: 1.18–2.33, *P* = .004), underscoring the role of arterial stiffness as an important clinical indicator for evaluating renal health (Table 3 and Fig. 3).

**Table 3:** Logistic regression analysis of risk factors for albuminuria.

Variables	Univariate			Multivariate		
	Odds ratio	95% CI	*P* value	Odds ratio	95% CI	*P* value
Age(y/o)	1.04	(1.03–1.05)	<.001	1.01	(0.99–1.02)	.36
Sex (men vs women)	1.15	(0.88–1.48)	.31	1.24	(0.93–1.65)	.14
WHtR*10	3.17	(2.55–3.94)	<.001	1.53	(1.18–1.98)	.001
MAP (mm Hg)	1.07	(1.06–1.07)	<.001	1.05	(1.03–1.06)	<.001
FPG (mmol/L)	1.39	(1.32–1.47)	<.001	1.26	(1.19–1.34)	<.001
TG/HDL	1.11	(1.06–1.17)	<.001	1.03	(0.99–1.07)	.16
baPWV ≧ 1400 cm/s	4.58	(3.50–5.99)	<.001	1.66	(1.18–2.33)	.004

baPWV = brachial-ankle pulse wave velocity, CI = confidence interval, FPG = fasting plasma glucose, HDL-C = high-density lipoprotein cholesterol, MAP = mean arterial pressure, TG = triglycerides, WHtR = waist-to-height ratio.

## 4. Discussion

### 4.1. Prevalence differences

The prevalence of albuminuria varies substantially across populations, with prior studies reporting rates of 9.4% in China, 8.2% in the United States, and 14.9% in Japan.^[18–20]^ In contrast, our study observed a lower prevalence of 4.96%, which may be attributed to the relatively younger age and overall healthier status of participants undergoing voluntary health checkups. Participants in such settings are also more likely to exhibit higher health literacy and engage in preventive health behaviors, further contributing to the lower observed rates. These differences underscore the importance of considering demographic and behavioral factors when interpreting albuminuria prevalence, and support the development of population-tailored screening strategies.

### 4.2. baPWV and albuminuria

This study contributes additional evidence supporting the association between arterial stiffness, as measured by baPWV, and renal impairment. Our results align with previous findings from Korean and Chinese cohorts that reported a positive association between elevated baPWV and albuminuria,^[21–23]^ and are consistent with longitudinal data from hypertensive and general Chinese populations in which higher baPWV predicted subsequent microalbuminuria or a faster decline in estimated GFR.^[24,25]^ Additionally, impaired kidney function has been associated with increased PWV in both longitudinal and cross-sectional analyses,^[26–28]^ raising the possibility of a reciprocal relationship between vascular and renal dysfunction, although causality remains to be determined.

### 4.3. Role of endothelial dysfunction

The glomerular filtration barrier is composed of the glycocalyx, glomerular endothelium, basement membrane, and podocytes, and functions to prevent the filtration of large proteins such as albumin.^[29]^ Disruption of this barrier, particularly via endothelial injury, has been implicated in increased permeability and albuminuria.^[30]^ The glycocalyx, a negatively charged proteoglycan-rich layer, is especially vulnerable to oxidative stress and inflammatory stimuli, leading to structural compromise and enhanced albumin leakage.^[31–33]^ These findings suggest a pivotal role for endothelial integrity in maintaining renal function.

### 4.4. Potential common mechanisms

Arterial stiffness is a multifactorial condition driven by changes in vascular structure and function, including chronic inflammation, extracellular matrix remodeling, and hemodynamic alterations.^[34,35]^ A growing body of evidence implicates endothelial dysfunction (particularly impaired nitric oxide bioavailability) as a key contributor to vascular stiffening.^[36,37]^ Our findings are in line with the hypothesis that endothelial injury not only mediates arterial stiffness but also contributes to glomerular dysfunction. This mechanistic overlap supports the notion that albuminuria and arterial stiffness may share a common endothelial-based pathophysiology.

### 4.5. Therapeutic implications

Interventions that restore endothelial function, including statins and angiotensin-converting enzyme inhibitors, have demonstrated efficacy in reducing arterial stiffness.^[38]^ Emerging therapies such as sodium-glucose cotransporter 2 inhibitors may further provide vascular and renal protection through anti-inflammatory and endothelial-preserving effects.^[39,40]^ In addition, non-pharmacologic strategies such as regular physical activity, dietary optimization, and smoking cessation remain essential components in preserving vascular health and preventing disease progression. Beyond nephrology and cardiology, noninvasive indices of arterial stiffness such as the cardio-ankle vascular index have also shown potential clinical utility in other specialties, including urological diseases, underscoring the broader clinical relevance of vascular stiffness assessment in routine care.^[41]^ These findings support early intervention strategies targeting endothelial function as a unified approach to mitigate both renal and cardiovascular risk.

### 4.6. Future research

Given the observed associations and plausible shared mechanisms, future research should aim to clarify the temporal and causal relationships between arterial stiffness and albuminuria. Prospective studies incorporating repeated measures of baPWV, albuminuria, and endothelial biomarkers will be critical to disentangle cause from consequence. A deeper understanding of these interactions may inform more precise therapeutic strategies and risk stratification approaches for patients vulnerable to both kidney and cardiovascular diseases.

## 5. Limitations

This study has several limitations that warrant consideration. First, the cross-sectional design precludes causal inferences between albuminuria and arterial stiffness. Longitudinal studies are needed to establish the temporality and causality of these associations. Second, our cohort consisted of health checkup participants who may represent individuals with higher health awareness and potentially lower risk profiles compared to the general population, thus limiting the generalizability of our findings. Third, although baPWV is a valid and convenient measure of arterial stiffness in clinical settings, it is not as extensively validated as carotid-femoral PWV, which is considered the gold standard. Therefore, the results may not be directly comparable to those of studies using different methodologies to assess arterial stiffness.

The study did not account for all potential confounders, such as dietary patterns, smoking status, physical activity levels, and genetic factors, all of which may influence both albuminuria and arterial stiffness. In addition, the use of a single ACR measurement may not accurately reflect persistent albuminuria; repeated assessments would provide a more reliable indication of CKD progression. Finally, our findings are based on a Chinese cohort, which may have specific genetic, environmental, and lifestyle characteristics affecting the prevalence and impact of albuminuria and arterial stiffness. Caution is therefore advised when extrapolating these results to other ethnic groups or populations.

Despite these limitations, our study provides valuable insights into the relationship between albuminuria and arterial stiffness, and highlights the potential utility of baPWV as a noninvasive tool for the early detection of CKD and cardiovascular risk stratification.

## 6. Conclusions

The present study demonstrated a significant association between albuminuria and arterial stiffness in a Chinese health checkup population, with baPWV identified as an independent marker of albuminuria. These findings suggest that baPWV is a valuable tool for detecting endothelial dysfunction and identifying individuals at risk for CKD and cardiovascular complications. Integrating baPWV into routine health checkups may enhance cardiovascular risk evaluation, while interventions targeting endothelial health could help prevent or slow disease progression. Future longitudinal studies are needed to confirm these cross-sectional results and clarify causal pathways.

## Author contributions

**Conceptualization:** Wei-Chung Yeh, Jau-Yuan Chen.

**Data curation:** Wei-Chung Yeh, Wen-Cheng Li, Jau-Yuan Chen.

**Methodology:** Jau-Yuan Chen.

**Supervision:** Mei-Wen Wang, Chen-Wei Hsu, Yu-Chung Tsao, Yu-Hsiang Lin, Jau-Yuan Chen.

**Writing – original draft:** Wei-Chung Yeh, Wen-Cheng Li.

**Writing – review & editing:** Jau-Yuan Chen.
